# Large nuclear vacuoles in spermatozoa negatively affect pregnancy rate in IVF cycles

**Published:** 2015-07

**Authors:** Shahin Ghazali, Ali Reza Talebi, Mohammad Ali Khalili, Abbas Aflatoonian, Navid Esfandiari

**Affiliations:** 1 *Research and Clinical Center for Infertility, Shahid Sadoughi University of Medical Sciences, Yazd, Iran.*; 2 *Division of Reproductive Endocrinology and Infertility, Department of Obstetrics and Gynecology, Geisel School of Medicine at Dartmouth, Lebanon, New Hampshire, USA.*

**Keywords:** *MSOME*, *Sperm head vacuole*, *IVF*, *ICSI*, *Pregnancy rate*

## Abstract

**Background:**

Recently, motile sperm organelle morphology examination (MSOME) criteria as a new real time tool for evaluation of spermatozoa in intracytoplasmic sperm injection (ICSI) cycles has been considered.

**Objective:**

The aim was to investigate the predictive value of MSOME in in vitro fertilization (IVF) in comparison to ICSI cycles and evaluation of the association between MSOME parameters and traditional sperm parameters in both groups.

**Materials and Methods:**

This is a cross sectional prospective analysis of MSOME parameters in IVF (n=31) and ICSI cycles (n=35). MSOME parameters were also evaluated as the presence of vacuole (none, small, medium, large or mix); head size (normal, small or large); cytoplasmic droplet; head shape and acrosome normality. In sub-analysis, MSOME parameters were compared between two groups with successful or failed clinical pregnancy in each group.

**Results:**

In IVF group, the rate of large nuclear vacuole showed significant increase in failed as compared to successful pregnancies (13.81±9.7vs7.38±4.4, respectively, p=0.045) while MSOME parameters were the same between successful and failed pregnancies in ICSI group. Moreover, a negative correlation was noticed between LNV and sperm shape normalcy. In ICSI group, a negative correlation was established between cytoplasmic droplet and sperm shape normalcy. In addition, there was a positive correlation between sperm shape normalcy and non-vacuolated spermatozoa.

**Conclusion:**

The high rate of large nuclear vacuoles in sperm used in IVF cycles with failed pregnancies confirms that MSOME, is a helpful tool for fine sperm morphology assessment, and its application may enhance the assisted reproduction technology success rates.

## Introduction

With introduction of intracytoplasmic sperm injection (ICSI), new era was introduced for treatment of male factor infertility (1). In this procedure, an immobilized sperm with normal morphology is injected into a mature oocyte. Since inception of ICSI, more attentions have been given to the recognition and selection of high quality spermatozoa to improve the outcome of assisted reproduction technology (ART) treatment. It is well known that sperm quality affects the quality of generated embryo (2). Sperm morphology has been shown as the best prognostic factor, among sperm parameters, to predict spontaneous pregnancy, intra-uterine insemination (IUI) and conventional in vitro fertilization (IVF) success rates (3-5). On the other hand, injection of spermatozoa with abnormal morphology is associated with lower fertilization and implantation rates (6). In addition, the sperm morphology is a powerful tool that can be used to determine the right ART method for treating infertility (7), is the best indicator of male fertility (8) and considered a sensitive prognostic tool in IUI and IVF (9).

The selection of sperm for ICSI is according to rough morphological characteristics. Meanwhile, the assessment of sperm morphology before ICSI is difficult because of sperm motility and not precise due to observation under low magnification. There may be some sperm morphological abnormalities that are not determined, when examined at routine magnification for ICSI (×200 or ×400) or even after fixation and staining (×1000). Recently several techniques have been introduced for evaluation of sperm morphology, including light microscopy, atomic force microscopy and electron microscopy. While these are not considered as real time techniques, Bartoov et al. (2001) developed a new tool for evaluation of non-fixed sperm morphology. In this technique, using a high power inverted light microscope with magnification of >×6000 sperm morphology is evaluated in real time more precisely and at the sub-cellular level named motile sperm organelle morphology examination (MSOME) (10). It has been shown that there is a relationship between normal morphology of spermatozoa determined by MSOME and fertilization rate, embryo quality, as well as implantation, pregnancy rates and chance of having normal child (2, 11-22). To the best of our knowledge, there have not been any studies regarding the comparison of MSOME criteria between IVF and ICSI cycles. We aimed to compare the sperm morphology at high magnification between cycles with successful and failed pregnancy in IVF as well as ICSI cases.

## Materials and methods

**Patients**

Sixty-six infertile couples were included from October 2013 to August 2014 in this cross sectional prospective study that were treated by IVF (n=31) or ICSI (n=35) at Research and Clinical Center for Infertility, Yazd, Iran. Third party assisted reproduction cycles, patients with less than three oocytes, spermatozoa with only round head or double tail, testicular or epididymal sperm, and maternal age of >40 years were excluded. The study was approved by the ethics committee of Yazd Reproductive Sciences institute. Signed informed consents were obtained from the patients.

**Semen collection and sperm preparation**

Semen samples were collected in sterile wide-mouth containers by masturbation following 2-5 days of abstinence. Semen analysis and preparation was performed according to standard world health organization (WHO) guidelines (23). Immediately after liquefaction, the semen samples were prepared by two layer (80/40) density gradient centrifugation method. The final pellet was resuspended in 0.5 ml pre-warmed equilibrated Ham’s F10 supplemented with 5 mg/ml human serum albumin and incubated at 37^°^C with 5% CO2 until use. Makler counting chamber was used for evaluation of sperm count and motility and sperm morphology was assessed by Diff-Quick staining method.

**Motile sperm organelle morphology examination (MSOME)**

Several 5µ microdroplets of polivinylpyrrolidone (8% PVP, Vitrolife, Goteborg, Sweden) were placed on a sterile glass bottom dish (WillCo-Dish, Amsterdam, Netherlands) and overlaid withmineraloil (Ovoil 100, Vitrolife, Goteborg, Sweden). Then, 1µ of prepared sperm suspension was added to a forementioned microdroplets. The inverted microscope (TE300; Nikon, Tokyo, Japan) equipped with high power differential interference contrast optics was used for MSOME (6600×). The image’s capture and video recording for further analysis was performed with software (OCTAX PolarAIDE; Octax). MSOME classification was defined according to Cassuto-Barak classification (2). Accordingly, the sperm morphology was evaluated based on the head shape, presence of vacuole in the nucleus and the head base. The score of each spermatozoon was determined as: (2×Head) + (3×Vacuole) + (Base). The scoring for each sperm cell ranged between 0 up to 6. Class I was high quality spermatozoa scored 4 to 6, Class II was medium quality spermatozoa scored 1 to 3, Class III was low quality sperm scored 0. We also defined MSOME parameters as follows: vacuoles (none, small: <4%, medium: 4≥ <15%, large: ≥15% of sperm head area, mix: having more than two vacuoles) (Figures I and II), head size (anterior-posterior diameter): normal: 5±0.2µm, small: ≤4.7µm, large: ≥5.3µm, head shape: normal: oval, abnormal: non-oval, cytoplasmic droplet: absence or presence (Figure III). At least 200 motile spermatozoa per patient were assessed, and each parameter was reported as percentage. The same researcher in a blind manner performed all sperm analysis, so inter observer variability was not assessed. MSOME parameters were compared between successful and failed pregnancy.

**ICSI and IVF**

The type of ART was selected for each case based of etiology of infertility, sperm parameters, female age, and patient’s history. IVF and ICSI techniques were performed as described elsewhere (24-25). Standard long protocol was used for controlled ovarian hyperstimulation which previously described (26). The injected oocytes after microinjection and conventionally inseminated COCs were evaluated 16-18 hrs later for presence of two pronuclei and two polar bodies to determine normal fertilization. Evaluations of embryo quality and embryo transfer were performed on day two. Embryo grading was previously described (27). Grade A and B embryos were considered as high quality embryos. Clinical pregnancy was confirmed after detection of fetal heart beat seven weeks after embryo transfer.

**Statistical analysis**

The data are presented as mean±SD and median (min-max) for quantitative and percentage for qualitative parameters. Shapiro-Wilk was used for evaluating normal distribution for quantitative data. Mann-Whitney U-test was applied in order to compare the MSOME parameters between IVF and ICSI groups. Chi-square test was used to compare proportions between two groups. Pearson test was performed for evaluation of correlation between different morphological parameters. The significant level was defined at p < 0.05.

## Results

There were no significant differences for patient’s age, sperm concentration, progressive motility, normal morphology, metaphase II oocytes, fertilized oocyte, formed embryos between successful and failed pregnancy cycles in IVF and ICSI groups (Table I and II).

Regarding MSOME parameters in IVF group, the data showed no significant difference for Cassuto-Barak classification, cytoplasmic droplet, and medium vacuole between successful and failed pregnancy cycles (Table III). However, the rate of LNV was significantly lower in successful pregnancies compared to the negative cycles in IVF group (7.38±4.4 vs 13.81±9.7, respectively, p=0.045), while this difference was not significant in ICSI group. Sperm shape and size were also the same between two groups. In ICSI group, only the rate of sperm shape normalcy was significantly higher in failed pregnancy compared to successful pregnancy (Table IV).

In IVF group, data showed a negative correlation between LNV and sperm shape normalcy (r=-0.48, p=0.006). There was also a positive correlation between the rate of combination of non-vacuole and small vacuole spermatozoa and sperm shape normalcy (r= 0.55, p=0.001). A significant positive correlation was also found between the rate of sperm normal head and normal shape(r=0.36, p=0.04) with progressive motility (r=0.38, p=0.03). In ICSI group, a negative correlation was established between cytoplasmic droplet and sperm shape normalcy (r=-0.34, p=0.04). A positive correlation between sperm shape normalcy and non-vacuolated spermatozoa (r=0.45, p=0.006), positive correlation between LNV and non-progressive motility (r=0.38, p=0.02), negative correlation between large head and normal shape (r=-0.43, p=0.009) were also found.

**Table I:** Demographic characteristics and outcomes in IVF cycles with successful and failed pregnancies

**Variables**	**Groups**		**p-value**
	**Successful pregnancy (n=8)**	**Failed pregnancy (n=16)**	
Male age (year) a	34.2±4.9	32.8±5.9	0.6
Female age (year) a	29.1±3.6	30.4±5	0.1
Sperm concentration (106 ml-1) b	98.4±22.7 105 (60-120)	97.7±42.7 89.5 (50-210)	0.2
Progressive motility (%)^b^	54.1±10.4 52 (38-68)	50.7±10 52.5 (27-70)	0.6
Normal morphology (%)^b^	16.2±5.1 20 (10-20)	15.7±6.4 15 (8-27)	0.8
Metaphase II oocyte^b^	13.6±5 12 (9-25)	9.7±4.8 11.5 (1-20)	0.2
Fertilized oocytes^b^	8.6±4.7 7.5 (5-19)	8±4.4 7.5 (1-20)	0.9
Formed embryos^b^	7.9±4.1 6.5 (5-17)	7.6±4.5 7 (1-20)	0.7
Transferred embryos^b^	2.4±0.5 2 (2-3)	2.1±0.5 2 (1-3)	0.4
Good quality embryos (%)*	62.5	46.7	0.6

a : Data are shown as mean±SD (Independent sample t-test).

b : Data are shown as mean±SD, median (min-max) (Man-Whitney U test).

*: Chi-square test

**Table II:** Demographic characteristics and outcome in ICSI cycles with successful and failed pregnancies

**Variables**	**Groups**		**p-value**
	**Successful pregnancy (n=7)**	**Failed pregnancy (n=23)**	
Male age (year) a	34.7±4.6	36.1±6	0.4
Female age (year) a	31±2.8	30.3±4.9	0.7
Sperm concentration (106 ml-1) b	87.1±59.7 80 (20-200)	67.9±32.9 60 (10-150)	0.4
Progressive motility (%) b	23.3±19.7 21 (0-53)	38.8±19.5 42 (1-71)	0.06
Normal morphology (%) b	8.1±9.3 5 (1-28)	12.6±8.4 12 (0-30)	0.1
Metaphase II oocyte^b^	10.6±5.1 9 (4-18)	8.52±4.8 7 (3-19)	0.3
Fertilized oocytes^b^	7.4±3.6 6 (3-12)	5.9±3.6 5 (2-16)	0.2
Formed embryos^b^	7.4±3.6 6 (3-12)	5±2.7 6 (3-12)	0.1
Transferred embryos^b^	2.4±0.5 2 (2-3)	2.3±0.4 2 (2-3)	0.8
Good quality embryos (%)*	85.7	43.5	0.08

a : Data are shown as mean±SD (Independent sample t-test).

b : Data are shown as mean±SD, median (min-max) (Man-Whitney U test).

*: Chi-square test

**Table III:** MSOME criteria and Cassuto-Barak classification in IVF group

**Parameters**	**Groups**		**p-value**
	**Successful pregnancy (n=8)**	**Failed pregnancy (n=16)**	
Class I *	7.4±3.5 7.5 (2-12)	8.9±7.3 7.5 (0-33)	1
Class II**	52.4±14.9 52 (23-76)	45.9±8.3 47 (25-56)	0.1
Class III***	40.4±16.7 38 (17-75)	45.2±11.7 45.5 (15-69)	0.1
Class I + Class II	59.7±16.8 62 (25-83)	54.7±11.7 54.5 (31-85)	0.1
Cytoplasmic droplet	20.5±6.6 20 (10-28)	23.9±10.4 21.5 (11-47)	0.4
Large vacuole	7.4±4.4 6 (1-15)	13.8±9.7 12 (3-42)	0.04
Medium vacuole	22.5±9.8 23.5 (8-39)	26.7±8.8 25.5 (15-48)	0.3
Small vacuole	28.6±5.6 28.5 (20-36)	20±8.6 17.5 (4-40)	0.01
Non-vacuole	18.6±13.4 14.5 (6-43)	16.7±10.4 12.5 (7-45)	0.9
Mixed vacuole	22.9±8.2 20.5 (13-38)	23.7±9.6 24.5 (9-37)	0.7
Normal head	55.9±13.4 55 (30-78)	54.2±8.6 56 (39-67)	0.6
Large head	3.5±4 3 (0-13)	3.8±3 4 (0-10)	0.4
Small head	39.4±17.6 43 (9-69)	42±8.6 42 (25-57)	0.7
Normal shape	28.7±9.8 26.5 (19-46)	31.1±13.9 32 (11-56)	0.6

Data are shown as mean±SD, median (min-max) (Man-Whitney U test).

*: Spermatozoa with high quality

**: Spermatozoa with medium quality

***: Spermatozoa with low quality

**Table IV:** MSOME criteria and Cassuto-Barak classification in ICSI group

**Parameters**	**Groups**		**p-value**
	**Successful pregnancy (n=7)**	**Failed pregnancy (n=23)**	
Class I*	2.4±2.8 2 (0-8)	4.5±3.2 4 (0-14)	0.08
Class II**	28.9±21.5 38 (3-55)	30.5±12.4 32 (8-65)	0.9
Class III***	68.7±23.8 60 (42-97)	65±14.7 62 (21-91)	0.9
Class I +Class II	31.3±23.8 40 (3-58)	35±14.7 38 (9-79)	0.9
Cytoplasmic droplet	32.9±19.2 25 (10-60)	25±10.5 22 (12-53)	0.3
Large vacuole	11.7±3.3 12 (6-16)	9.7±7 8 (2-35)	0.1
Medium vacuole	27.3±9.5 27 (15-42)	21.9±7.2 21 (9-37)	0.1
Small vacuole	19.9±9.4 19 (6-30)	23.3±6 24 (11-33)	0.3
Non-vacuole	7.7±8.1 5 (1-24)	11.8±5.5 11 (0-24)	0.07
Mixed vacuole	33.4±8.7 31 (25-50)	32.8±8.9 32 (14-50)	0.9
Normal head	26±14.9 28 (0-46)	34.6±18.7 31 (8-82)	0.4
Large head	6.4±9.9 2 (0-27)	8.7±5.3 8 (0-18)	0.1
Small head	67.6±15.8 62 (53-100)	56.1±18.3 60 (16-90)	0.2
Normal shape	7.4±6.6 7 (0-20)	15.3±7.5 15 (1-30)	0.01

Data are shown as mean±SD, median (min-max) (Man-Whitney U test).

*: Spermatozoa with high quality

**: Spermatozoa with medium quality

***: Spermatozoa with low quality

**Figure 1:**
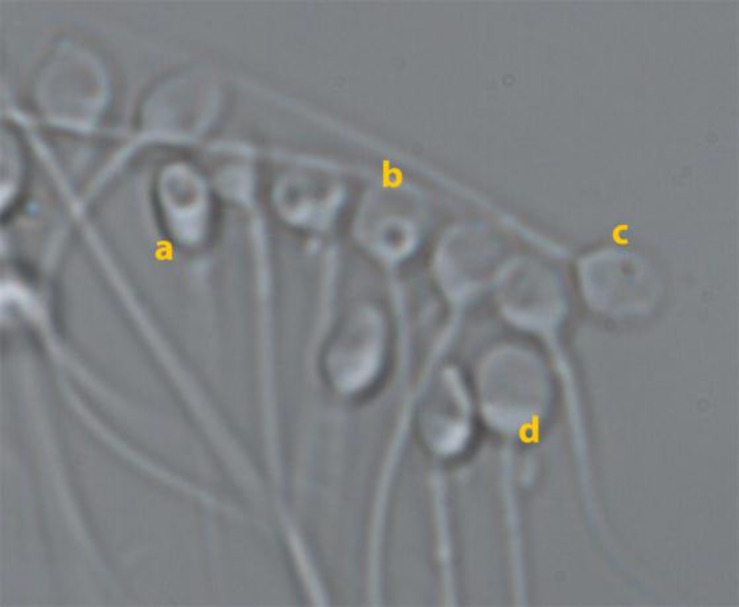
Spermatozoa at high magnification (6600×).a and b: spermatozoa with large nuclear vacuole (≥15% of head area), c: spermatozoon with medium nuclear vacuole (4≥ <15% of head area), d: non-vacuole spermatozoon

**Figure 2:**
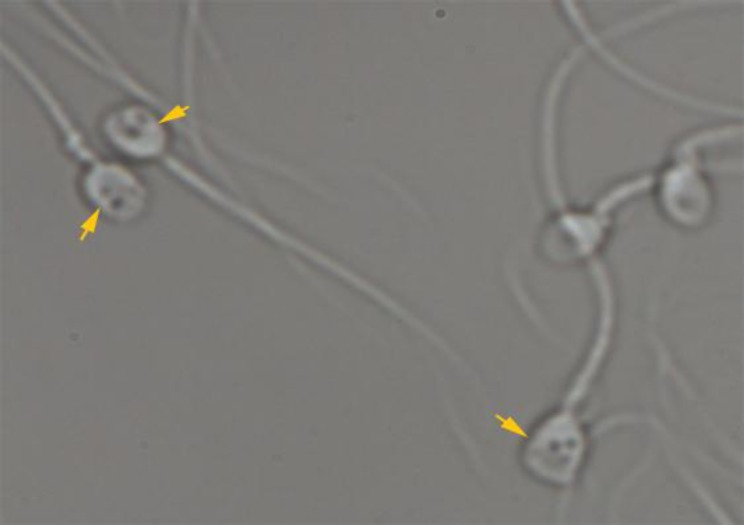
Spermatozoa at high magnification (6600×). The arrows the spermatozoa with mix vacuoles (having more than two vacuoles).

**Figure 3:**
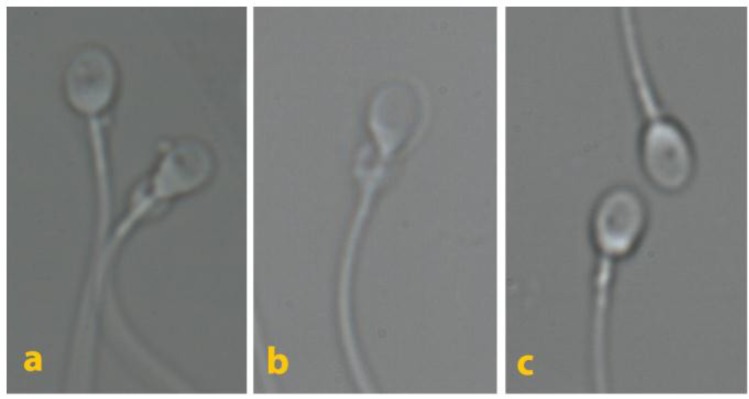
Spermatozoa at high magnification (6600×).a and b: spermatozoa with cytoplasmic droplet, c: spermatozoa having no cytoplasmic droplet

## Discussion

Application of MSOME has not become a routine practice in ART clinics yet. In general, MSOME can evaluate the acrosome, post-acrosomal lamina, neck, tail, mitochondria and nucleus, in which the sperm nucleus is the most important organelle (28). Chromatin content can also be assessed by MSOME, as it was shown that the homogenous chromatin contains no more than one vacuole that occupies less than four percent of nuclear area (11). In addition to determination of overall view of sperm structure, MSOME can detect sperm nuclear vacuoles in more details. Previous studies have shown that nuclear vacuoles have detrimental effect on fertilization, embryo development and pregnancy outcome (29-30), while we did not find any correlation between MSOME parameters and fertilization and embryo development, the rate of LNVs were significantly higher in patients with successful pregnancies as compared to the patients with failed pregnancies.

One of the proposed indications of MSOME is its predictive value for pregnancy in IUI (31). Evaluation of sperm morphology at high magnification for IVF cycles is recommended, because sperm morphology is an important factor for IVF success. To our knowledge, this is the first report regarding the MSOME criteria compared between IVF and ICSI cycles. Recently, De Vos et al. (2013) analyzed 330 semen samples from ICSI cycles. They stated that 18.1% of spermatozoa had normal shape with no vacuole as compared to our results of 11.8% in failed pregnancy cycles (32). Berkovitz et al. (2006) indicated that prevalence of LNV in spermatozoa from patients undergoing ICSI is 30-40%, which is higher compared to our results (11.7%) (29). One probable cause for this discrepancy would be due to different definitions of LNV that are used in different studies. We defined the LNV as the vacuole occupying>15% of nuclear area; while, others have defined a vacuole area >4%, >13%, > 25% or> 50% of head area (29, 33-35). This needs to be standardized if this technique wants to be integrated in routine practice in infertility diagnosis and treatment. We have also defined medium size vacuole (4≥ and <15% of head area) in order to standardize definition of vacuole. Our results also showed that the percent of abnormal sperm head size and shape, spermatozoa with cytoplasmic droplet and class III (according to Cassuto-Barak classification) were significantly higher in men who had abnormal semen parameters (ICSI group) compared to normal semen parameters (IVF group) (data not shown). Perdrix et al. (2012) evaluated MSOME criteria and conventional semen analysis in a population of 440 males. They found that the sperm head width and area as well as nuclear vacuoles number and area are significantly higher in men with abnormal semen (n=331) as compared to those with normal semen parameters (n=109) (36).

Paternal age is considered as one of the main factors that affects sperm morphology. A recent study showed that prevalence of large and small vacuoles will increase with increasing paternal age (37). In our study, paternal age between successful and failed cycles both in IVF and ICSI were the same. Regarding the association between morphology evaluated with MSOME and conventional semen analysis, it was shown that there was no significant correlation between normal morphology by WHO and the ones defined by MSOME (26.1±7.2 vs 2.9±0.5, respectively) (11). While other investigators have found that MSOME might be more strict than Tygerberg criteria (8).

Our data showed that the presence of LNV affects the normalcy of sperm shape whereas there was positive correlation between the rate of small and non-vacuole and sperm shape normalcy. It might be concluded that LNV should be considered as a definite parameter for evaluation of sperm shape. Interestingly, our results showed that there is significant correlation between sperm normal head and progressive motility. Theoretically, based on hydrodynamic rules, shape of sperm head is the most determinant factor for motility and in case of sperm head abnormality, sperm motility could be impaired. The impact of LNV on progressive motility is our another important finding that shows the other detrimental effect of LNV is inhibition of sperm motility.

## Conclusion

Although, MSOME is a time consuming method with no cost benefit, and demanding experienced operator, it can provide more details regarding fine sperm morphology in a real time manner. MSOME can be used as a predictive factor, and in case of high LNV rate, the cycle may not be considered for IVF, but substituted with ICSI. Therefore, MSOME should be considered as a non-invasive adjunct technology assisting ART performance.

## Note

***This article extracted from Ph.D. thesis. (Shahin Ghazali)***
